# A New Frequentist Implementation of the Daniels and Hughes Bivariate Meta‐Analysis Model for Surrogate Endpoint Evaluation

**DOI:** 10.1002/bimj.70048

**Published:** 2025-03-19

**Authors:** Dan Jackson, Michael Sweeting, Robbie C. M. van Aert, Sylwia Bujkiewicz, Keith R. Abrams, Wolfgang Viechtbauer

**Affiliations:** ^1^ Statistical Innovation Group, AstraZeneca Cambridge UK; ^2^ Tilburg University Tilburg the Netherlands; ^3^ Biostatistics Research Group Department of Population Health Sciences University of Leicester Leicester UK; ^4^ Department of Statistics and Warwick Medical School University of Warwick Coventry UK; ^5^ Maastricht University Maastricht the Netherlands

**Keywords:** bias correction, maximum likelihood estimation, meta‐regression, multivariate meta‐analysis, surrogate endpoint

## Abstract

Surrogate endpoints are used when the primary outcome is difficult to measure accurately. Determining if a measure is suitable to use as a surrogate endpoint is a challenging task and a variety of meta‐analysis models have been proposed for this purpose. The Daniels and Hughes bivariate model for trial‐level surrogate endpoint evaluation is gaining traction but presents difficulties for frequentist estimation and hitherto only Bayesian solutions have been available. This is because the marginal model is not a conventional linear model and the number of unknown parameters increases at the same rate as the number of studies. This second property raises immediate concerns that the maximum likelihood estimator of the model's unknown variance component may be downwardly biased. We derive maximum likelihood estimating equations to motivate a bias adjusted estimator of this parameter. The bias correction terms in our proposed estimating equation are easily computed and have an intuitively appealing algebraic form. A simulation study is performed to illustrate how this estimator overcomes the difficulties associated with maximum likelihood estimation. We illustrate our methods using two contrasting examples from oncology.

## 1 Introduction

Surrogate endpoints are used when the primary endpoint is difficult to measure accurately, for example when little information is accessible or information may only become available in the longer term. A common example in the context of oncology trials is the use of progression‐free survival (PFS) as a surrogate endpoint for overall survival (OS; Belin et al. 2020). Determining if an outcome is suitable to use as a surrogate endpoint is a challenging task in practice, despite the criteria that have been proposed for this purpose (e.g., Baker 2018; Daniels and Hughes 1997; Prentice 1989).

We focus on the situation where evidence from multiple trials is available and a statistical assessment of trial‐level surrogacy is required. A number of approaches have been developed that require individual patient data (IPD; Burzykowski et al. 2001; Burzykowski et al. 2021; Buyse 2000; Dai and Hughes, 2012; Molenberghs et al. 2008). We agree with Li and Meredith (2003) that models using IPD are to be preferred when these data are available because associations between patient outcomes can then be quantified. However, it is typically the case that only aggregate (or summary level) data are available to the analyst and we address this common situation. Meta‐analytic approaches focus on the association between treatment effects and so, in the framework of Taylor and Elston (2009), assess “level one” evidence for surrogacy. In general, other methods will be needed to assess evidence at their second (associations between outcomes) and third (biological plausibility) levels.

Bujkiewicz et al. (2017) provide an accessible overview of methods for meta‐analysis, that can be used to assess if an outcome is a suitable surrogate endpoint, requiring only aggregate level study information. One of these methods is the model proposed by Daniels and Hughes (1997), where a linear regression is assumed for the true effects across studies. Advantages of the Daniels and Hughes (1997) model are that it does not require the estimation of a between‐study correlation parameter, which can be challenging (Burke et al. 2018), and it does not assume that the surrogate treatment effects are exchangeable (Bujkiewicz et al. 2017). However this model is, in some respects, unusual. For example, the marginal model is not a conventional linear model. Furthermore, it uses a separate fixed effect for the candidate surrogate treatment effect from each study, so that the number of unknown parameters increases at the same rate as the number of studies. These two features make the Daniels and Hughes (1997) model difficult to satisfactorily fit in a frequentist framework, where the main challenge relates to estimating its unknown variance component. This is because the presence of a separate fixed effect for each study can result in notable downward bias in maximum likelihood estimates of variance parameters (Jackson, Law, et al. 2018), analogously to the well‐known Neyman and Scott (1948) problem.

Extensions of the Daniels and Hughes (1997) model are possible, for example, where there is more than one potential surrogate (Pozzi et al. 2016) or multiple treatment classes (Papanikos et al. 2020), and we return to this in the discussion. There has therefore been considerable methodological development since Daniels and Hughes (1997) and this model is gaining traction in Health Technology Assessment (HTA). For example, it is described in detail in NICE Technical Support Document 20 (Bujkiewicz, Achana, et al. 2019). We therefore anticipate that the application of the Daniels and Hughes model to increase. Our position is that it is useful to have Bayesian and frequentist estimation methods available to us. Our work fills an important void by providing, to the best of our knowledge, the first frequentist implementation of the Daniels and Hughes model.

The main competitor to the Daniels and Hughes model is the bivariate random‐effects meta‐analysis model (Jackson et al. 2011; Bujkiewicz et al. 2017; Bujkiewicz, Jackson, et al. 2019) that has been extended to the network meta‐analysis setting (Achana et al. 2014; Bujkiewicz, Achana, et al. 2019; Efthimiou et al., 2014; Jackson, Bujkiewicz, et al. 2018). This is a linear model where the number of parameters does not depend on the number of studies, so that this model is more amenable to frequentist methods. However it requires the estimation of the between‐study correlation and assumes that the surrogate treatment effects are exchangeable. Hence, the Daniels and Hughes and bivariate random‐effects meta‐analysis models are diametrically opposites in terms of these advantages and disadvantages. However, both these models acknowledge that the surrogate treatment effects are estimated with uncertainty. A wide variety of estimation methods for the between‐study covariance matrix are available for the bivariate random‐effects meta‐analysis model but they are not immediately applicable to the Daniels and Hughes model. For example, the theory for the restricted maximum likelihood estimator (REML; Jennrich and Schluchter 1986), that helps to correct for the downward bias of maximum likelihood estimates of variance components (Jackson et al. 2011), assumes a linear model. Moment‐based estimators for bivariate random‐effects meta‐analysis (Jackson et al. 2013) have not been developed for the Daniels and Hughes model, and estimators of this type possess no optimality properties. Conventional meta‐regression (Thompson and Sharp 1999) is another competing method (Bujkiewicz et al. 2017) that instead includes the treatment effect on the surrogate endpoint as a covariate.

Daniels and Hughes (1997) propose fitting their model in the Bayesian framework, which circumvents the estimation difficulties encountered when using frequentist methods, but comes at the price of sensitivity to prior distribution specification. In particular, the conventional univariate random‐effects model for meta‐analysis has been found to be sensitive to the prior distribution used for the between‐study variance parameter (Lambert et al. 2005). This can also be anticipated to be the case for the unknown variance component of the Daniels and Hughes model. The aim of this paper is to develop frequentist estimation methods for this model and so avoid concerns related to prior sensitivity. We achieve this by considering maximum likelihood estimation and then using the resulting estimating equations to derive a bias adjusted estimator of the unknown variance component. This estimate can then be used in a bias adjusted frequentist analysis using the Daniels and Hughes (1997) model that addresses the potential bias in the corresponding maximum likelihood estimator.

The rest of the paper is set out as follows. In Section 2, we present the Daniels and Hughes model. In Section 3, we develop maximum likelihood estimating equations and we derive a bias adjusted estimator of the unknown variance component in Section 4. In Section 5, we perform a small‐scale simulation study that provides proof of concept that our adjusted estimator overcomes the problems associated with using maximum likelihood estimation in conjunction with the Daniels and Hughes model. In Section 6, we apply our methodology to two contrasting examples and we conclude with a short discussion.

## 2 The Daniels and Hughes Model

The Daniels and Hughes (1997) bivariate meta‐analysis model for surrogate endpoint evaluation assumes that each study provides two estimates (with corresponding standard errors and within‐study correlation): the estimated treatment effect on the clinical outcome of interest and the surrogate treatment effect. For example, the estimated treatment effect on the clinical outcome of interest could be an estimated log hazard ratio comparing two treatment groups for OS and the surrogate treatment effect could be the corresponding log hazard ratio for PFS. The overall aim is to determine if the surrogate is viable (see Section 2.4 below).

Let n be the number of studies included in the analysis. For the ith study, let $\theta_{i}$ and $\gamma_{i}$, i=1,2,…n, be the true treatment effect on the clinical outcome of interest, and on the surrogate, respectively. Let ${\hat{\theta }}_{i}$ and ${\hat{\gamma }}_{i}$ be the corresponding estimates that are usually available or can be extracted from published papers or reports, and so are aggregate level information available to the analyst.

### 2.1 The Within‐Study Model

Daniels and Hughes (1997) assume the standard within‐study model for bivariate meta‐analysis (their eq. (1); Jackson et al. 2011), and so assume that

(1) $$\left(\begin{array}{c} {\hat{\theta }}_{i}\\ {\hat{\gamma }}_{i} \end{array}\right)∼N\left(\left(\begin{array}{c} \theta_{i}\\ \gamma_{i} \end{array}\right),\left(\begin{array}{cc} \sigma_{i}^{2} & \rho_{i}\sigma_{i}\delta_{i}\\ \rho_{i}\sigma_{i}\delta_{i} & \delta_{i}^{2} \end{array}\right)\right).$$

Model (1) describes how the distributions of ${\hat{\theta }}_{i}$ and ${\hat{\gamma }}_{i}$ depend on the true $\theta_{i}$ and $\gamma_{i}$. We treat the within‐study covariance matrix in model (1) as fixed and known. Typically the within‐study variances, $\sigma_{i}^{2}$ and $\delta_{i}^{2}$, will be known but the within‐study correlations $\rho_{i}$ between ${\hat{\theta }}_{i}$ and ${\hat{\gamma }}_{i}$ are unlikely to be directly reported in aggregate level data. We agree with Riley (2009) that using appropriate values for the within‐study correlations is an important consideration. A variety of approaches are available that produce suitable values of $\rho_{i}$ (Bujkiewicz et al. 2013; Bujkiewicz, Achana, et al. 2019; Riley et al. 2008; 2015; Wei and Higgins 2013) and we explore two possibilities in our examples below.

### 2.2 The Between‐Studies Model

Between studies, Daniels and Hughes (1997) assume that

(2) $$\theta_{i}|\gamma_{i}∼N\left(\alpha +\beta \gamma_{i},\tau^{2}\right).$$

Model (2) is the between‐studies model because it states the assumptions made about the relationships between the true treatment effects on the two outcomes across trials. Equation (2) is different to the conventional between‐study model for random‐effects bivariate meta‐analysis (Jackson et al. 2011; their eq. (2)) that instead assumes that $\left(\theta_{i},\gamma_{i}\right)$ follows a bivariate normal distribution. It is therefore model (2) that distinguishes the Daniels and Hughes model from the bivariate random‐effects meta‐analysis model. The parameter $\tau^{2}$ is the (conditional on $\gamma_{i}$) between‐study variance of $\theta_{i}$ and is the focus of attention in much of the work that follows. Model (2) follows from the bivariate random‐effects model but does not make the assumption of bivariate normality.

### 2.3 The Marginal Model

The implied marginal model, from models (1) and (2), is

(3) $$\left(\begin{array}{c} {\hat{\theta }}_{i}\\ {\hat{\gamma }}_{i} \end{array}\right)∼N\left(\left(\begin{array}{c} \alpha +\beta \gamma_{i}\\ \gamma_{i} \end{array}\right),\left(\begin{array}{cc} \sigma_{i}^{2}+\tau^{2} & \rho_{i}\sigma_{i}\delta_{i}\\ \rho_{i}\sigma_{i}\delta_{i} & \delta_{i}^{2} \end{array}\right)\right).$$

In the frequentist framework, inferences are usually based on the marginal model. Hence we take model (3) to be the Daniels and Hughes model. The Daniels and Hughes model makes normality assumptions both within (model 1) and between (model 2) studies. Methods for meta‐analysis that make fewer normality assumptions are in general available (Jackson and White 2018) and we return to the possibility of relaxing these assumptions in the discussion. We assume that each pair of $\left({\hat{\theta }}_{i},{\hat{\gamma }}_{i}\right)$ is independent.

### 2.4 Assessing Surrogacy

All three parameters α, β, and $\tau^{2}$ in model (3) are useful when determining if the putative surrogate endpoint is suitable as a good predictor of clinical benefit measured by the clinical outcome of interest, which is discussed by Daniels and Hughes (1997). Briefly, α=0, β≠0, and $\tau^{2}=0$ are taken to be indicative of a perfect surrogate relationship. If α=0, then no treatment effect for the surrogate corresponds to no treatment effect for the outcome of clinical interest. If β≠0, then a treatment effect for the surrogate is associated with a treatment effect for this outcome; if the same directional effects indicate treatment benefit for the surrogate and outcome of clinical interest, then β>0 is suggestive of a surrogate relationship. Finally, if $\tau^{2}=0$, then the treatment effect on the final clinical outcome can be predicted perfectly given the effect on the surrogate endpoint. All three parameters in model (2) should be used to assess surrogacy. The $\gamma_{i}$ are usually regarded as nuisance parameters.

Hence $\hat{\alpha }\approx 0$ and ${\hat{\tau }}^{2}\approx 0$, estimated with small uncertainty, and a confidence or credible interval for β that does not include zero, indicate that the surrogate is considered suitable. However, the extent to which different departures from this are tolerable when declaring surrogacy is an open question. We return to this issue in the discussion.

### 2.5 Challenges for Frequentist Inference

As explained in the introduction, model (3) presents two main challenges for frequentist estimation. First, there is a separate parameter $\gamma_{i}$ for each study, so that the number of parameters increases at the same rate as the number of studies. Hence the maximum likelihood estimator of $\tau^{2}$ may be badly downwardly biased (Jackson, Law, et al. 2018). Second, model (3) is a nonlinear model because it contains the term $\beta \gamma_{i}$ in the linear predictor for ${\hat{\theta }}_{i}$, which is not linear in terms of the model parameters. One implication of this is that the theory of restricted maximum likelihood (REML; Jennrich and Schluchter 1986) does not apply.

Despite concerns about downward bias for the maximum likelihood estimate of $\tau^{2}$, we will begin by deriving maximum likelihood estimating equations for all model parameters. These estimating equations will then be used to motivate a bias adjusted estimator of $\tau^{2}$.

## 3 Maximum Likelihood Estimating Equations

The likelihood function is the product of densities of the bivariate normal probability density function from model (3), where each study contributes one such term. Maximum likelihood estimating equations can be derived using the properties of the normal distribution.

### 3.1 Estimating Equation for $\gamma_{i}$

We use ${\hat{\gamma }}_{i,MLE}$, i=1,…n, to denote the *model‐based* maximum likelihood estimate of $\gamma_{i}$. Conceptually, ${\hat{\gamma }}_{i,MLE}$ and ${\hat{\gamma }}_{i}$ are estimates of the same quantity $\gamma_{i}$. The difference however is that ${\hat{\gamma }}_{i,MLE}$ uses information from all studies, assuming the meta‐analysis model (3), whereas ${\hat{\gamma }}_{i}$ is aggregate level data used in analysis and uses information only from the ith study.

The likelihood function is the product of probability density functions implied by model (3), so that the only terms in this function that contain $\gamma_{i}$ are from the ith study. When differentiating the log‐likelihood function with respect to $\gamma_{i}$, only the logarithm of the bivariate normal density contribution for the ith study remains. Reparameterizing so that ${\hat{\theta }}_{i}^{*}={\hat{\theta }}_{i}-\alpha$, model (3) becomes

(4) $$\left(\begin{array}{c} {\hat{\theta }}_{i}^{*}\\ {\hat{\gamma }}_{i} \end{array}\right)∼N\left(C_{\left(\beta ,1\right)}\gamma_{i},\left(\begin{array}{cc} \sigma_{i}^{2}+\tau^{2} & \rho_{i}\sigma_{i}\delta_{i}\\ \rho_{i}\sigma_{i}\delta_{i} & \delta_{i}^{2} \end{array}\right)\right),$$

where $C_{\left(\beta ,1\right)}$ is a column vector of length 2 where the first entry is β and the second is one. Noting that only the contribution from the ith study remains when differentiating the log‐likelihood function with respect to $\gamma_{i}$, ${\hat{\gamma }}_{i,MLE}$ satisfies the maximum likelihood estimating equation for $\gamma_{i}$ from model (4) where all other parameters are replaced by their maximum likelihood estimates. Model (4) is a weighted linear regression model, so that the standard theory for this type of model can be used to derive the resulting maximum likelihood estimating equation for $\gamma_{i}$.

Let ${\hat{W}}_{i}$ be the inverse of the covariance matrix in (4) where $\tau^{2}$ is replaced by its maximum likelihood estimate, ${\hat{\tau }}^{2}$. Standard theory for weighted linear regression models results in the estimating equation 

(5) $${\hat{\gamma }}_{i,MLE}={\left(C_{\left(\hat{\beta },1\right)}^{T}{\hat{W}}_{i}C_{\left(\hat{\beta },1\right)}\right)}^{-1}C_{\left(\hat{\beta },1\right)}^{T}{\hat{W}}_{i}\left(\begin{array}{c} {\hat{\theta }}_{i}^{*}\\ {\hat{\gamma }}_{i} \end{array}\right),$$

where all estimates are maximum likelihood estimates. Estimating Equation (5) could be used as part of an iterative scheme to estimate all parameters, where estimates at the previous step are used on the right‐hand side to produce the maximum likelihood estimate of $\gamma_{i}$ at the current step on the left. Equation (5) is the basis for n estimating equations, because there is one such equation for each $\gamma_{i}$.

### 3.2 Estimating Equations for α and β

We factorize the contributions to the likelihood from the ith study resulting from the bivariate normal model (3) as contribution from the marginal density of ${\hat{\gamma }}_{i}∼N\left(\gamma_{i},\delta_{i}^{2}\right)$ and the contribution from the conditional density

$${\hat{\theta }}_{i}|{\hat{\gamma }}_{i}∼N\left(\alpha +\beta \gamma_{i}+\left(\rho_{i}\sigma_{i}/\delta_{i}\right)\left({\hat{\gamma }}_{i}-\gamma_{i}\right),\sigma_{i}^{2}\left(1-\rho_{i}^{2}\right)+\tau^{2}\right).$$

To simplify notation, let $\mu_{i}\left({\hat{\gamma }}_{i}\right)=\alpha +\beta \gamma_{i}+\left(\rho_{i}\sigma_{i}/\delta_{i}\right)\left({\hat{\gamma }}_{i}-\gamma_{i}\right)$ and $v_{i}^{2}=\sigma_{i}^{2}\left(1-\rho_{i}^{2}\right)$. Then the conditional distribution of ${\hat{\theta }}_{i}|{\hat{\gamma }}_{i}$ above becomes

(6) $${\hat{\theta }}_{i}|{\hat{\gamma }}_{i}∼N\left(\mu_{i}\left({\hat{\gamma }}_{i}\right),v_{i}^{2}+\tau^{2}\right).$$

Model (6) is a weighted linear regression with an offset. Estimating equations are therefore easily derived, as explained in Appendix A.

### 3.3 Estimating Equation for $\tau^{2}$

The marginal model ${\hat{\gamma }}_{i}∼N\left(\gamma_{i},\delta_{i}^{2}\right)$ does not depend on $\tau^{2}$. Hence, when differentiating the log‐likelihood function with respect to $\tau^{2}$, only terms from the conditional distributions of ${\hat{\theta }}_{i}|{\hat{\gamma }}_{i}$ from model (6) contribute. When omitting terms that do not include $\tau^{2}$, to within a constant the log‐likelihood is

(7) $$\ell \left(\tau^{2}\right)=\sum_{i=1}^{n}\left(\log\left(v_{i}^{2}+\tau^{2}\right)+{\left({\hat{\theta }}_{i}-\mu_{i}\left({\hat{\gamma }}_{i}\right)\right)}^{2}/\left(v_{i}^{2}+\tau^{2}\right)\right).$$

Differentiating Equation (7) with respect to $\tau^{2}$, setting the resulting expression to zero, and replacing all parameters with their maximum likelihood estimates gives the estimating equation

(8) $$\sum_{i=1}^{n}\left(1/\left(v_{i}^{2}+{\hat{\tau }}^{2}\right)-{\left({\hat{\theta }}_{i}-{\hat{\mu }}_{i}\left({\hat{\gamma }}_{i}\right)\right)}^{2}/\left({\left(v_{i}^{2}+{\hat{\tau }}^{2}\right)}^{2}\right)\right)=0,$$

where ${\hat{\mu }}_{i}\left({\hat{\gamma }}_{i}\right)=\hat{\alpha }+\hat{\beta }{\hat{\gamma }}_{i,MLE}+\left(\rho_{i}\sigma_{i}/\delta_{i}\right)\left({\hat{\gamma }}_{i}-{\hat{\gamma }}_{i,MLE}\right)$. Rearranging Equation (8) quickly provides the estimating equation

(9) $${\hat{\tau }}^{2}=\frac{\sum_{i=1}^{n}\left({\left({\hat{\theta }}_{i}-{\hat{\mu }}_{i}\left({\hat{\gamma }}_{i}\right)\right)}^{2}-v_{i}^{2}\right)/{\left(v_{i}^{2}+{\hat{\tau }}^{2}\right)}^{2}}{\sum_{i=1}^{n}1/{\left(v_{i}^{2}+{\hat{\tau }}^{2}\right)}^{2}}.$$

Equation (9) is analogous to eq. (3b) from Thompson and Sharp (1999). We truncate ${\hat{\tau }}^{2}=0$ if the estimate from Equation (9) is negative.

We have now derived maximum likelihood estimating Equations ((5), (9), (A2), (A3)), where two of these equations are in Appendix A, for all model parameters. Note that parameter estimates appear on the right‐hand sides of these estimating equations, so that they may be used in iterative scheme to fit the model; they are not closed‐form solutions that can be used to compute the maximum likelihood estimates.

#### 3.3.1 Assumptions Required for Estimating Equation (9) for $\tau^{2}$ to Be Unbiased

Before considering bias correction, it is insightful to understand the conditions under which estimating Equation (9) is unbiased. Suppose that all model parameters, α, β, $\tau^{2}$, and all $\gamma_{i}$, were known and are used to evaluate the right‐hand side of estimating Equation (9). Then we replace the estimates in Equation (9) with their corresponding true parameter values and this estimating equation becomes

(10) $${\hat{\tau }}^{2}=\frac{\sum_{i=1}^{n}\left({\left({\hat{\theta }}_{i}-\mu_{i}\left({\hat{\gamma }}_{i}\right)\right)}^{2}-v_{i}^{2}\right)/{\left(v_{i}^{2}+\tau^{2}\right)}^{2}}{\sum_{i=1}^{n}1/{\left(v_{i}^{2}+\tau^{2}\right)}^{2}}.$$

We can evaluate the expectation of (10) as

(11) $$E\left[{\hat{\tau }}^{2}\right]=\frac{\sum_{i=1}^{n}\left(E\left[E\left[{\left({\hat{\theta }}_{i}-\mu_{i}\left({\hat{\gamma }}_{i}\right)\right)}^{2}|{\hat{\gamma }}_{i}\right]\right]-v_{i}^{2}\right)/{\left(v_{i}^{2}+\tau^{2}\right)}^{2}}{\sum_{i=1}^{n}1/{\left(v_{i}^{2}+\tau^{2}\right)}^{2}}.$$

From the conditional model (6), in Equation (11), we have $E\left[{\left({\hat{\theta }}_{i}-\mu_{i}\left({\hat{\gamma }}_{i}\right)\right)}^{2}|{\hat{\gamma }}_{i}\right]=v_{i}^{2}+\tau^{2}$, where $v_{i}^{2}$ and $\tau^{2}$ are both treated as constants. Hence $E\left({\hat{\tau }}^{2}\right)=\tau^{2}$, so that ${\hat{\tau }}^{2}$ is then unbiased. If we knew the true parameter values, there would of course be no need to estimate $\tau^{2}$, but this does clarify that the form of the estimating Equation (9) is a good starting point.

## 4 Bias Adjusted Estimating Equation for $\tau^{2}$

The main concern is that the presence of an unknown fixed effect $\gamma_{i}$ for each study may result in downward bias for the maximum likelihood estimate of $\tau^{2}$. We now repeat the analysis in Section 3.3.1 but where we take into account the fact that the estimates ${\hat{\gamma }}_{i,MLE}$, rather than the true values $\gamma_{i}$, are used in the estimating equation for $\tau^{2}$ in Equation (9). However, we will treat $\hat{\alpha }$, $\hat{\beta ,}$ and ${\hat{\tau }}^{2}$ as fixed constants when evaluating the right‐hand side of Equation (9). This is because the main difficulties for the estimation of $\tau^{2}$ are associated with the number of unknown $\gamma_{i}$ increasing at the same rate as the number of studies. This approach deals with the most serious problem in an analytically tractable way.

Our proposed bias adjusted estimate of $\tau^{2}$ is a modified version of the estimating Equation (9) given by

(12) $${\hat{\tau }}_{adjusted}^{2}=\frac{\sum_{i=1}^{n}\left({\left(1-b_{i}\right)}^{-2}{\left({\hat{\theta }}_{i}-{\hat{\mu }}_{i}\left({\hat{\gamma }}_{i}\right)\right)}^{2}-v_{i}^{2}\right)/{\left(v_{i}^{2}+{\hat{\tau }}^{2}\right)}^{2}}{\sum_{i=1}^{n}1/{\left(v_{i}^{2}+{\hat{\tau }}^{2}\right)}^{2}},$$

where $b_{i}=\left(\hat{\beta }-\rho_{i}\sigma_{i}/\delta_{i}\right)w_{1i}$; $w_{1i}$ is the first entry of the row vector ${\left(C_{\left(\hat{\beta },1\right)}^{T}{\hat{W}}_{i}C_{\left(\hat{\beta },1\right)}\right)}^{-1}C_{\left(\hat{\beta },1\right)}^{T}{\hat{W}}_{i}$.

The mathematical result required to derive the bias adjusted estimating Equation (12) is derived in Appendix B. We motivate this estimating equation by taking iterated expectations in (12), in the same way as in Section 3.3.1, but now using Equation (B4) from Appendix B to evaluate the inner expectations. This calculation shows that the expected value of ${\hat{\tau }}_{adjusted}^{2}$ in estimating Equation (12) is $\tau^{2}$, so that estimating Equation (12) removes bias in the maximum likelihood estimate of $\tau^{2}$ associated with the need to estimate the $\gamma_{i}$. We truncate ${\hat{\tau }}_{adjusted}^{2}=0$ if the estimate from Equation (12) is negative.

### 4.1 Evaluating the Bias Correction Terms

The key quantities are the “bias adjustment terms” $b_{i}=\left(\hat{\beta }-\rho_{i}\sigma_{i}/\delta_{i}\right)w_{1i}$ in estimating Equation (12). This is because if all $b_{i}=0$, then no adjustment to the maximum likelihood estimate ${\hat{\tau }}^{2}$ from Equation (9) is made. We can explicitly compute $w_{1i}$ by computing ${\left(C_{\left(\hat{\beta },1\right)}^{T}{\hat{W}}_{i}C_{\left(\beta ,1\right)}\right)}^{-1}C_{\left(\hat{\beta },1\right)}^{T}{\hat{W}}_{i}$ and extracting the first entry. This is straightforward because ${\hat{W}}_{i}$ is the inverse of the 2×2 covariance matrix in (4); when computing this inverse using the adjoint method the determinant cancels in the calculation of ${\left(C_{\left(\hat{\beta },1\right)}^{T}{\hat{W}}_{i}C_{\left(\beta ,1\right)}\right)}^{-1}C_{\left(\hat{\beta },1\right)}^{T}{\hat{W}}_{i}$, which simplifies the calculation, and we obtain

$$w_{1i}=\frac{\hat{\beta }\delta_{i}^{2}-\rho_{i}\sigma_{i}\delta_{i}}{\sigma_{i}^{2}+{\hat{\tau }}^{2}+{\hat{\beta }}^{2}\delta_{i}^{2}-2\hat{\beta }\rho_{i}\sigma_{i}\delta_{i}}.$$

Hence

(13) $$b_{i}=\frac{\left(\hat{\beta }-\rho_{i}\sigma_{i}/\delta_{i}\right)\left(\hat{\beta }\delta_{i}^{2}-\rho_{i}\sigma_{i}\delta_{i}\right)}{\sigma_{i}^{2}+{\hat{\tau }}^{2}+{\hat{\beta }}^{2}\delta_{i}^{2}-2\hat{\beta }\rho_{i}\sigma_{i}\delta_{i}}=\frac{{\left(\hat{\beta }\delta_{i}-\rho_{i}\sigma_{i}\right)}^{2}}{{\left(\hat{\beta }\delta_{i}-\rho_{i}\sigma_{i}\right)}^{2}+{\hat{\tau }}^{2}+v_{i}^{2}}.$$

Equation (13) shows that $0\leq b_{i}<1$, so that in Equation (12) we have $1\leq {\left(1-b_{i}\right)}^{-2}<\infty$. Hence positive scaling terms that cannot result in a smaller estimate of $\tau^{2}$ are applied to the squared residuals when using the bias adjusted estimator (12).

If $\hat{\beta }=\rho_{i}\sigma_{i}/\delta_{i}$, then $b_{i}=0$ and no adjustment is made for the ith study. From Equation (2), β is the gradient of the between‐study regression of $\theta_{i}$ on $\gamma_{i}$. Furthermore, in the within‐study model (1) for the ith study, $\rho_{i}\sigma_{i}/\delta_{i}$ is the gradient associated with ${\hat{\gamma }}_{i}$ in $E({\hat{\theta }}_{i}|{\hat{\gamma }}_{i})$. We will therefore refer to $\rho_{i}\sigma_{i}/\delta_{i}$ and β as the “within‐study gradient,” and “between‐study gradient,” respectively. Conceptually, if the estimated within and between‐study gradients associated with the clinical outcome of interest on the surrogate are identical, no adjustment is made, via the bias adjustment terms $b_{i}$, when estimating $\tau^{2}$ using maximum likelihood. Furthermore, the adjustment becomes larger as the magnitude of the difference between these two gradients increases. The bias adjustment terms therefore have a clear and intuitive interpretation. Our results indicate that maximum likelihood estimation of $\tau^{2}$ can be expected to perform well when the dependence of the clinical outcome on the surrogate is in this sense similar within and between studies. The bias adjustment terms $b_{i}$ are functions of parameter estimates, so that they may also be conceptualized as estimates, but we refrain from presenting them as ${\hat{b}}_{i}$ because they do not estimate parameters of inferential interest.

## 5 Simulation Study

We performed a small‐scale simulation study to provide proof of concept that maximum likelihood estimates of $\tau^{2}$ can be badly biased downwards, and further that the corresponding bias adjusted estimator addresses this problem.

### 5.1 Simulation Study Design

We used n=20 studies throughout, so that the Daniels and Hughes model can be adequately estimated while assuming a plausible sample size. We assumed that the true $\gamma_{i}$ are uniformly distributed from −5 to 5. We assumed that the within‐study variances, $\sigma_{i}^{2}=\delta_{i}^{2}=0.05,0.1,\text{…}1.00$, are the same and fixed for each simulated data set. For simplicity, we took $\sigma_{i}^{2}=\delta_{i}^{2}$ for all studies.

We varied the other four model parameters, using three values of β=0,0.4,0.8 and similarly three different values of $\rho_{i}=0,0.4,0.8$ (where the same $\rho_{i}$ was assumed for all studies). From the theory derived in Section 4, maximum likelihood estimation of $\tau^{2}$ should perform well in terms of bias when ρ=β=0, 0.4, or 0.8 because then, in the absence of bias in $\hat{\beta }$, $\hat{\beta }\approx \rho_{i}\sigma_{i}/\delta_{i}$ so that $b_{i}\approx 0$. Otherwise the bias adjusted estimator should provide an improvement. We used three different values of $\tau^{2}=0,0.5,1$. If $\tau^{2}=0$, then, because ${\hat{\tau }}^{2}\geq 0$, the conventional maximum likelihood estimator can only exhibit positive bias. Hence $\tau^{2}=0$ was considered, not because this is necessarily plausible, but because it was of interest to assess the extent that the bias adjusted estimator may exacerbate this. We used two different values of α=0,0.25 but we did not expect this to have much impact on the estimation performance. We therefore examined 3×3×3×2=54 different scenarios. One thousand simulations were used in each setting, so that in total our methods were applied to 54,000 simulated data sets.

### 5.2 Computational Details

Conventional maximum likelihood estimation was performed numerically where $\log(\tau^{2})$, and all other unknown parameters, may take any real value. The resulting $\log({\hat{\tau }}^{2})$ was then exponentiated to provide ${\hat{\tau }}^{2}>0$, where very large negative values of $\log({\hat{\tau }}^{2})$ correspond to ${\hat{\tau }}^{2}=0$. All numerical maximum likelihood estimates were then substituted into the estimating Equations ((5), (9), (A2), (A3)) to confirm that the correct solutions had been found. An advantage of using the estimating equations is that they can be used to remove all doubt that numerical optimizers have correctly converged; there is a separate $\gamma_{i}$ for each study, so the dimension of the optimization problem can be high, and convergence may be difficult to correctly identify in practice.

Standard errors were computed as the inverse of the Hessian matrix, where this matrix was obtained numerically. Computing the standard error of $\log({\hat{\tau }}^{2})$ in this way however presents problems when ${\hat{\tau }}^{2}=0$, because small changes in very large and negative values of $\log({\hat{\tau }}^{2})$ result in negligible differences in the likelihood function. If this occurs, then all partial derivatives with respect to $\log(\tau^{2})$ will be calculated to be zero, resulting in a Hessian that is not invertible. To avoid this difficulty, the generalized matrix inverse of the Hessian was used, which is numerically equivalent to constraining $\tau^{2}=0$ in the analysis when ${\hat{\tau }}^{2}=0$. A more computationally intensive alternative is to use methods based on the profile likelihood and we return to this possibility in the discussion. Wald‐type 95% confidence intervals for α and β were computed using their point estimates, standard errors, and normal approximations.

An adjusted analysis was then performed constraining $\tau^{2}={\hat{\tau }}_{adjusted}^{2}$ and performing maximum likelihood estimation for the other model parameters, so that the bias adjusted estimate of $\tau^{2}$ was used instead of the maximum likelihood estimator. Estimating Equations ((5), (A2), (A3)) were used, with this constraint, to confirm that numerical optimizers had correctly converged. Using the estimated between‐study variance as the true value in this way is a common approximation in meta‐analysis (Jackson and White 2018). Although the accuracy of this approximation may be questionable, for example, in situations where the number of studies is small, the greater concern is that a conventional maximum likelihood–based analysis may result in severe downward bias for $\tau^{2}$.

The main outcomes of interest were the biases of the estimates of α,β, and $\tau^{2}$, and the coverage probabilities of the 95% confidence intervals of α and β. Calculating confidence intervals for $\tau^{2}$ is a complex issue even under the univariate random‐effects model for meta‐analysis and we return to this issue in the discussion.

We also compared our frequentist analyses to a more conventional Bayesian analysis. Here “vague,” and independent, prior distributions of $\gamma_{i},\alpha ,\beta ∼N\left(0,1000\right)$ were used. In general the prior for $\tau^{2}$ can be expected to be influential (Lambert et al. 2005). Here we followed Bujkiewicz, Achana, et al. (2019) and used the prior τ∼U(0,2), that is, a uniform prior for the between‐study standard deviation. To make the Bayesian results more comparable with our frequentist analyses, we used the same values of $\rho_{i}$ in our modeling, instead of using prior distributions for the $\rho_{i}$ that may instead be used to allow for the possibility that these values are unknown. Our Bayesian analyses are merely intended to be representative, so that we can compare our frequentist methods to a typical type of Bayesian analysis. Posterior means, and coverage probabilities of 95% percentile–based credible intervals, can then be used to provide comparisons with frequentist biases and coverage probabilities. Posterior distributions of $\tau^{2}$ can be skew and biases were also computed using the posterior median. Burn ins of 1000 Markov chain Monte Carlo iterations were used. A further 10,000 iterations were used to make inferences with a thinning frequency of two.

### 5.3 Results

The full results are shown in the Supporting Information. No concerns relating to bias when estimating α were encountered in any setting or using any estimation method (see the Supporting Information).

**FIGURE 1:**
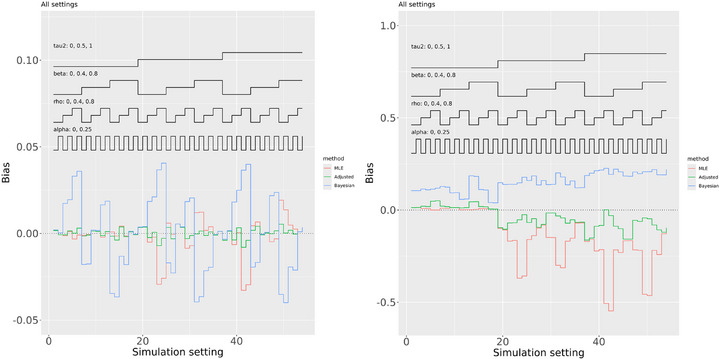
Bias across all scenarios when estimating β (left) and $\tau^{2}$ (right). Results for the maximum likelihood estimator (MLE) are shown in red, the bias adjusted (Adjusted) estimator are shown in green, and Bayesian (posterior mean) estimators are shown in blue.

The largest biases when estimating β were produced by the Bayesian analysis, and the adjusted analysis also resulted in noticeably smaller biases than conventional maximum likelihood estimation (Figure 1, left). A closer examination (see the Supporting Information) revealed that all three methods performed more similarly in scenarios where $\beta =\rho_{i}\sigma_{i}/\delta_{i}=\rho_{i}$. From our analysis in Section 3, we know that this criterion has important implications for the maximum likelihood estimation of $\tau^{2}$. It appears that it may also have implications for Bayesian analyses that also use the likelihood function. We should interpret this finding cautiously, because simulation study results may not generalize to other settings, but the observation that our proposed adjusted frequentist method performed best in terms of bias when estimating β is an important finding.

The Bayesian analysis consistently resulted in positive bias when estimating $\tau^{2}$ (Figure 1, right). This can be explained by the prior τ∼U(0,2) that gives considerable probability to larger values of $\tau^{2}$ than explored in our simulation study. This bias was reduced by instead using the posterior median of $\tau^{2}$ (see Supporting Information). The standard maximum likelihood analysis resulted in slight positive bias when estimating $\tau^{2}$ when the true value was zero, as expected, and the bias adjusted method slightly increased this (Figure 1, right). However standard likelihood–based estimation resulted in large negative bias when the true $\tau^{2}>0$ and the adjusted method greatly reduced, but did not completely remove, this bias. A closer examination (see the Supporting Information) revealed that the bias adjustment made little difference in the scenarios where $\beta =\rho_{i}\sigma_{i}/\delta_{i}=\rho_{i}$, for which the downward bias of the maximum likelihood estimate of $\tau^{2}$ was much less severe, as expected. We conclude that the estimation of $\tau^{2}$ is challenging but our adjusted method greatly reduces the problems resulting from using maximum likelihood estimation and avoids concerns related to prior sensitivity in Bayesian analyses.

**FIGURE 2:**
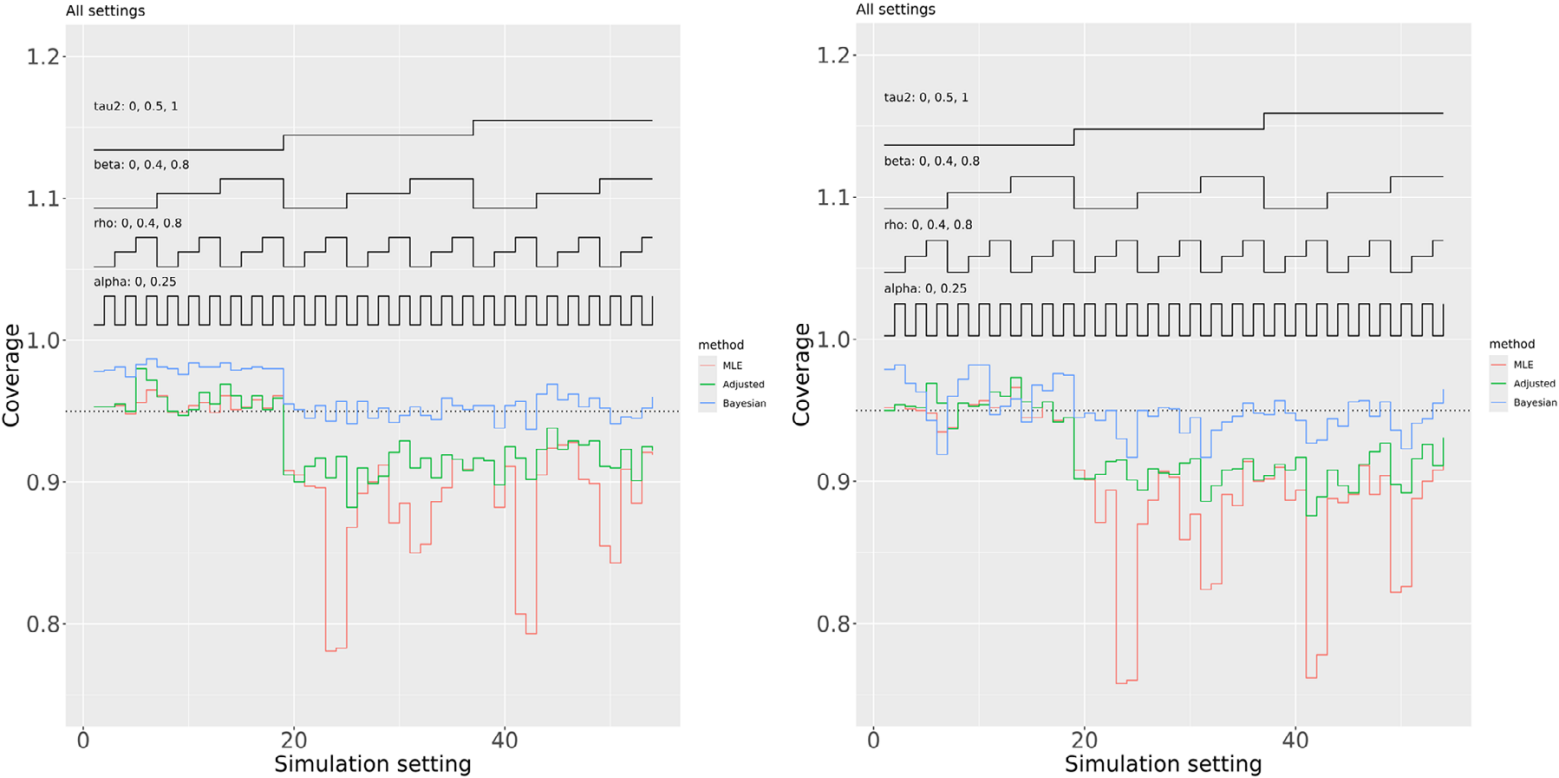
Coverage probabilities for α (left) and β (right). Results for the maximum likelihood estimator (MLE) are shown in red, the bias adjusted (Adjusted) estimator are shown in green, and Bayesian (percentile based) estimators are shown in blue.

The results for the coverage probabilities of confidence and credible intervals are shown in Figure 2. The Bayesian analysis generally performed well in terms of the coverage probability of 95% credible intervals for α and β, where these probabilities were close to 0.95 when $\tau^{2}>0$ and overcoverage was observed when $\tau^{2}=0$. The conventional maximum likelihood analysis resulted in severe under coverage when $\tau^{2}>0$, for example, as low as under 0.8. The lowest adjusted analysis coverage probabilities were instead around 0.9, which is a considerable improvement. The overall impression is that the bias adjustment greatly reduces the downward bias of the maximum likelihood estimation of $\tau^{2}$, and so avoids the severe under coverage of the confidence intervals for α and β, but does not entirely eliminate this. When $\tau^{2}=0$, the frequentist methods generally resulted in slight overcoverage for α but this was not evident for β. The two frequentist methods can only result in positive bias for $\tau^{2}$ when this is zero, so that overcoverage for the other parameters might then be anticipated, but fully accommodating the uncertainty in many $\gamma_{i}$ is likely to counteract this intuition. Ad hoc methods for increasing the coverage probability of confidence intervals, for example, by using the t distribution to compute them, might be worth considering but any concrete proposal for this would need to be carefully justified.

To summarize, the proposed “bias adjusted method” performs better than conventional maximum likelihood estimation by greatly reducing its main problems. Bayesian estimation performed best in terms of coverage probabilities for α and β but raises concerns about sensitivity to prior distributions. The two frequentist methods, and the adjusted method in particular, performed better in terms of bias when estimating β. No method performed best in all respects but our position is that this simulation study indicates that our proposed bias adjusted method is, at the very least, a viable alternative to more conventional Bayesian estimation of the Daniels and Hughes model.

## 6 Examples

### 6.1 Example 1: Antiangiogenic Therapies for Metastatic Colorectal Cancer

**FIGURE 3:**
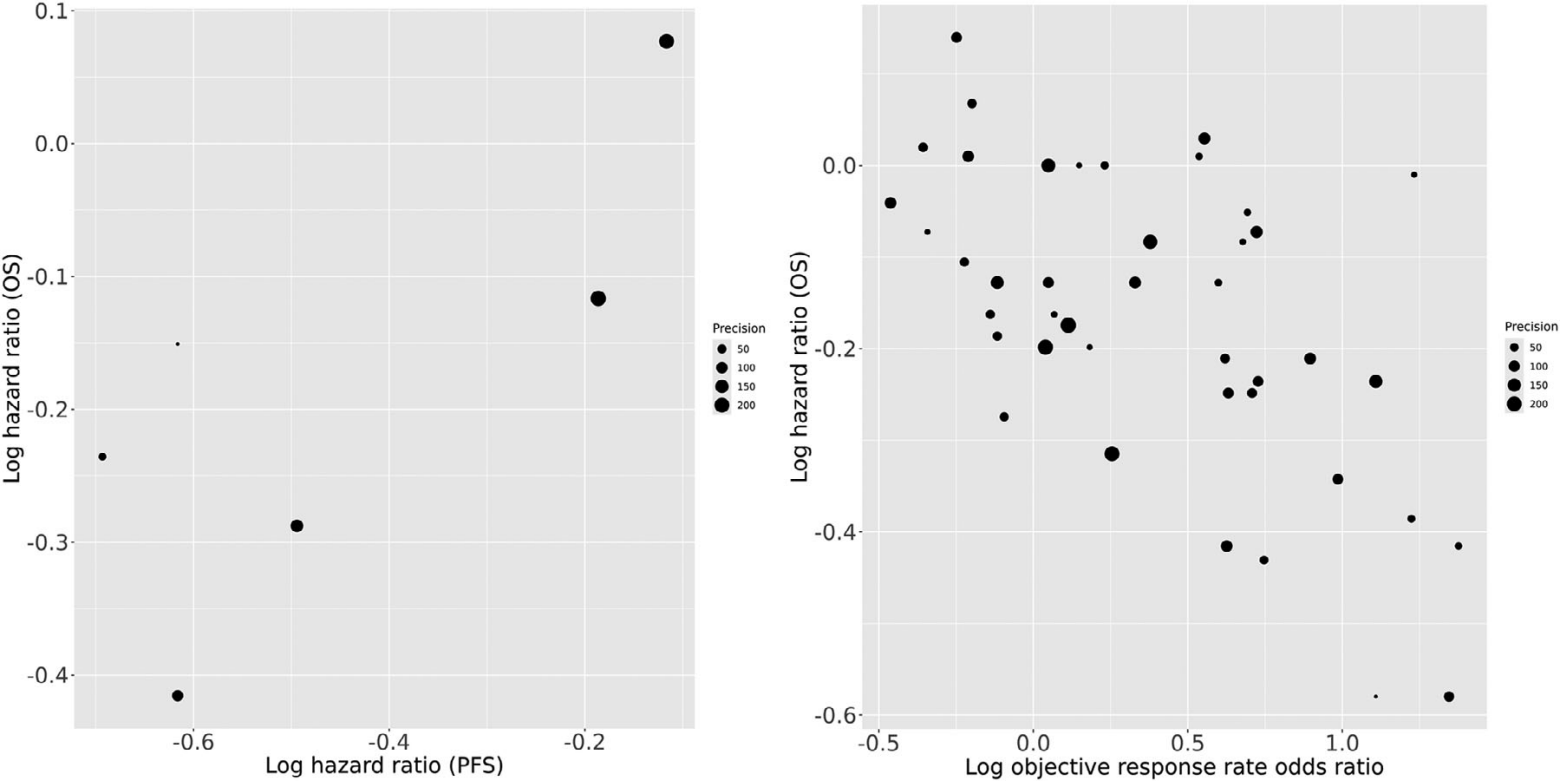
Scatter plots for example 1 (left, Section 6.1) and example 2 (right, Section 6.2).

The systematic review “Anti‐angiogenic therapies for metastatic colorectal cancer” (Wagner et al. 2009) contains six studies that report hazard ratios for PFS and OS. A scatter plot of these six pairs of estimates (log hazard ratios) are shown in Figure 3 (left). A within‐study correlation of $\rho_{i}=0.513$ is available for one of the studies (Elia et al. 2020) but for the others this is unknown. We will perform an analysis assuming that that the within‐study correlations are similar across studies and so use $\rho_{i}=0.513$ for all studies. Our analyses will explore if PFS is a potential surrogate endpoint for OS at the study level.

The results are shown in Table 1, where the same Bayesian method described in Section 5 was used for both this and the subsequent example. In addition to presenting posterior means and percentile based credible intervals (mean, %), as in the simulation study in Section 5, we also show posterior medians and highest probability density (HPD)‐based credible intervals. The adjusted and unadjusted frequentist estimates are ${\hat{\tau }}^{2}=0$ so that these two methods give the same results. This is because the six “within‐study gradients,” $\rho_{i}\sigma_{i}/\delta_{i}$, take values in the interval [0.453,0.623]. These values are are not sufficiently numerically dissimilar to the $\hat{\beta }=0.776$ for the bias adjustment to instead provide ${\hat{\tau }}_{adjusted}^{2}>0$. Although 95% credible intervals for $\tau^{2}$ are shown in Table 1, the corresponding confidence intervals are omitted because the best way to compute them is left as further work. We return to this issue in the discussion. The Bayesian (mean %) and (median, HPD) results for α and β in Table 1 are similar but the results for $\tau^{2}$ are notably different, and indicate that the posterior distribution of $\tau^{2}$ is skew. This is not surprising because there are just six pairs of estimates contributing to the analysis.

**TABLE 1:** Parameter estimates for example 1. Both frequentist analyses result in ${\hat{\tau }}^{2}=0$ and so give the same results. 95% confidence and credible intervals are shown in parentheses for α and β, and 95% credible intervals are also shown in parentheses for $\tau^{2}$.

Estimation method	α	β	$\tau^{2}$
Frequentist (both)	0.102 (−0.025, 0.229)	0.776 (0.421, 1.130)	0
Bayesian (mean, %)	0.078 (−0.270, 0.401)	0.678 (−0.132, 1.369)	0.030 (0.000, 0.204)
Bayesian (median, HPD)	0.081 (−0.274, 0.395)	0.688 (−0.093, 1.405)	0.008 (0.000, 0.125)

Our frequentist analyses support the results from the Bayesian analysis that PFS may be a suitable surrogate endpoint for OS. In particular, they reinforce the conclusion that $\tau^{2}$ is small, so that the variation in the true treatment effects on OS is explained by the corresponding treatment effects on PFS. Credible intervals for $\tau^{2}$ are wide, however, so this finding should be treated with caution. Both types of the analysis provide $\hat{\alpha }\approx 0$, which is also supportive of surrogacy. The point estimates of β are also similar but the Bayesian analysis has resulted in much wider interval estimation. In an example such as this, where the number of studies is small, the Bayesian analysis can reasonably claim to better describe model uncertainty. The Bayesian credible intervals for β include zero, whereas the frequentist analyses exclude this.

The overall impression is that more evidence is required because confidence and, in particular, credible intervals are wide, but PFS may be a suitable surrogate endpoint for OS. Our adjusted analysis indicates that concerns relating to the downward bias of the maximum likelihood estimate of $\tau^{2}$ is not an issue for this example.

### 6.2 Example 2: Objective Response Rate and Survival‐Based Endpoints in First‐Line Advanced Non–Small Cell Lung Cancer

Goring et al. (2022) recently performed a systematic review and meta‐analysis, “Correlations between objective response rate and survival‐based endpoints in first‐line advanced non‐small cell lung Cancer: A systematic review and meta‐analysis,” that includes 42 pairs of estimates. Here we reanalyze the data in their supplementary table A11 to explore whether the objective response rate is a potential surrogate endpoint for OS. A scatter plot of the 42 pairs of estimates (log objective response rate odds ratios and log OS hazard ratios) is shown in Figure 3 (right). The within‐study correlations are unknown but can be expected to be negative, because we assume that increase in response rate implies a reduction in the mortality rate. Hence we will perform a sensitivity analysis where we take $\rho_{i}=-0.1,-0.3,-0.5,-0.7,-0.9$ for all studies. The values $\rho_{i}=-0.1$ and $\rho_{i}=-0.9$ are probably implausibly small and large, respectively, but it is of interest to assess the extent to which inferences are sensitive to the assumed within‐study associations between the treatment effects on the potential surrogate endpoint and the final clinical outcome.

The results are shown in Table 2. The two frequentist methods give similar results for $\rho_{i}=-0.1,-0.3,-0.5$, where the bias adjusted estimates of $\tau^{2}$ are only slightly larger than the maximum likelihood estimates. This is because the “within‐study gradients,” $\rho_{i}\sigma_{i}/\delta_{i}$, take values in the intervals [−0.073,−0.046], [−0.220,−0.137], and [−0.366,−0.228], for these three values of $\rho_{i}$ respectively, that are not dissimilar to the corresponding maximum likelihood estimates of β (Table 2). However for $\rho_{i}=-0.7,-0.9$, the bias adjustment results in a much larger estimate of $\tau^{2}$ and provides results that are in better agreement with the Bayesian analysis. This is especially evident for $\rho_{i}=-0.9$ where the maximum likelihood estimate of $\tau^{2}$ is zero, whereas the corresponding bias adjusted estimate is ${\hat{\tau }}_{adjusted}^{2}=0.014$. The “within‐study gradients,” $\rho_{i}\sigma_{i}/\delta_{i}$, take values in the intervals [−0.513,−0.319] and [−0.659,−0.410] for $\rho_{i}=-0.7$ and $\rho_{i}=-0.9$, respectively, that are notably different from the corresponding maximum likelihood estimates of β. Our proposed method has therefore had considerable impact for the last two values of $\rho_{i}$ in our sensitivity analysis, where we observe better agreement with the Bayesian analysis when using our bias adjusted method. The Bayesian (mean, %) and (median, HPD) results are in much better agreement than the first example, as expected because many more pairs of estimates contribute to the analysis.

**TABLE 2:** Parameter estimates for example 2. 95% confidence and credible intervals are shown in parentheses for α and β, and 95% credible intervals are also shown in parentheses for $\tau^{2}$.

Estimation method	$\rho_{i}$	α	β	$\tau^{2}$
Frequentist (unadjusted)	−0.1	−0.088 (−0.137, −0.039)	−0.216 (−0.306, −0.126)	0.003
Frequentist (adjusted)	−0.1	−0.088 (−0.140, −0.036)	−0.214 (−0.308, −0.121)	0.004
Bayesian (mean, %)	−0.1	−0.097 (−0.148, −0.044)	−0.189 (−0.277, −0.103)	0.005 (0.000, 0.014)
Bayesian (median, HPD)	−0.1	−0.097 (−0.147, −0.044)	−0.188 (−0.278, −0.104)	0.004 (0.000, 0.012)
Frequentist (unadjusted)	−0.3	−0.094 (−0.143, −0.044)	−0.196 (−0.286, −0.107)	0.004
Frequentist (adjusted)	−0.3	−0.094 (−0.143, −0.044)	−0.196 (−0.286, −0.107)	0.005
Bayesian (mean, %)	−0.3	−0.095 (−0.149, −0.042)	−0.193 (−0.279, −0.102)	0.006 (0.001, 0.015)
Bayesian (median, HPD)	−0.3	−0.095 (−0.148, −0.041)	−0.193 (−0.279, −0.102)	0.005 (0.000, 0.013)
Frequentist (unadjusted)	−0.5	−0.100 (−0.150, −0.051)	−0.175 (−0.263, −0.086)	0.005
Frequentist (adjusted)	−0.5	−0.100 (−0.150, −0.050)	−0.176 (−0.265, −0.087)	0.006
Bayesian (mean, %)	−0.5	−0.093 (−0.143, −0.041)	−0.195 (−0.276, −0.110)	0.007 (0.001, 0.016)
Bayesian (median, HPD)	−0.5	−0.093 (−0.142, −0.040)	−0.195 (−0.276, −0.110)	0.006 (0.001, 0.014)
Frequentist (unadjusted)	−0.7	−0.112 (−0.160, −0.064)	−0.142 (−0.230, −0.054)	0.004
Frequentist (adjusted)	−0.7	−0.106 (−0.159, −0.052)	−0.157 (−0.249, −0.064)	0.009
Bayesian (mean, %)	−0.7	−0.092 (−0.143, −0.042)	−0.194 (−0.277, −0.111)	0.009 (0.003, 0.018)
Bayesian (median, HPD)	−0.7	−0.092 (−0.142, −0.041)	−0.194 (−0.275, −0.110)	0.008 (0.002, 0.017)
Frequentist (unadjusted)	−0.9	−0.151 (−0.193, −0.109)	−0.033 (−0.115, 0.048)	0
Frequentist (adjusted)	−0.9	−0.110 (−0.169, −0.050)	−0.144 (−0.243, −0.044)	0.014
Bayesian (mean, %)	−0.9	−0.091 (−0.141, −0.040)	−0.194 (−0.274, −0.114)	0.010 (0.004, 0.020)
Bayesian (median, HPD)	−0.9	−0.091 (−0.141, −0.040)	−0.194 (−0.272, −0.112)	0.010 (0.004, 0.018)

The overall impression is that our results support the conclusion from Goring et al. (2022) that objective response rate is associated with OS in this trial level meta‐analysis (β<0). Furthermore, estimates of $\tau^{2}$ are small. Estimates of α are not large but confidence and credible intervals do not include zero, which casts doubt on the use of the objective response rate as a surrogate for OS.

## 7 Conclusions

We have developed a new frequentist estimation method for fitting the Daniels and Hughes bivariate meta‐analysis model. The proposed estimation method is entirely based on this model but is otherwise fundamentally different to the current Bayesian implementation. Perhaps the main advantage of our approach is avoiding the need to specify prior distributions. Justifying particular priors for unknown variance components is challenging and in general inferences can be expected to be sensitive to this. The existing Bayesian approach, however, also has advantages. For example, it more naturally lends itself to decision analysis. We propose our estimation method as an alternative to, rather than a replacement of, a Bayesian analysis. A by‐product of our work is that we are able to clarify the circumstances where a standard maximum likelihood based analysis is appropriate and so when more conventional frequentist analyses are feasible.

More sophisticated statistical modeling is possible, for example, by allowing more than one type of potential surrogate, multiple treatment classes, and/or considering models that make fewer normality assumptions. For example, alternative within‐study models could be used to better describe binary outcome data and more sophisticated between‐study regression models be used for the studies' true treatment effects. Another important avenue for future research is to investigate the best methods for computing confidence intervals for the between‐study variance, for which profile likelihood based confidence intervals may prove to be a good solution. A wide variety of methods are available for this under the univariate random‐effects model (Veroniki et al. 2016). Extending some or all of these to the Daniels and Hughes model would be valuable. However this can be anticipated to be challenging because of the bias we have found in the corresponding maximum likelihood point estimate. More extensive simulation and empirical studies would also be highly worthwhile. This could include comparing the performance of methods based on different statistical models, for example the random‐effects bivariate meta‐analysis model and meta‐regression.

Three model parameters have been used to make inferences about whether a potential surrogate endpoint is suitable but more guidance concerning how to assess this would be valuable. We have adopted the approach of considering each of these parameters in turn, but the development of alternative statistical procedures, for example, that consider these parameters simultaneously may be worthwhile. For example, Daniels and Hughes (1997) used Bayes factors to test for associations. We have treated the $\gamma_{i}$ as nuisance parameters but study specific effects may also be of interest (van Aert et al. 2021). Other statistical outputs are also often of interest, for example, prediction intervals (van Aert et al. 2021) and $I^{2}$ statistics (Higgins and Thompson 2002). We leave the best way to compute quantities such as these as another avenue for further work.

To summarize, our work provides a new way to fit the Daniels and Hughes bivariate meta‐analysis model. We have applied our methodology, and a more conventional Bayesian analysis, to two examples. We have also performed a simulation study. Computing codes, in R, for both the examples and the simulation study are provided in the Supporting Information. To the best of our knowledge, our methods provide the first frequentist implementation of the Daniels and Hughes model. This model is gaining traction and so our work fills an important void in the current state of statistical science.

## Conflicts of Interest

Sylwia Bujkiewicz is a member of the NICE Decision Support Unit (DSU) and the NICE Guidelines Technical Support Unit (TSU). She has served as a paid consultant, providing methodological advice in relevant area, to NICE, Roche, RTI Health Solutions, and IQVIA and has received payments for educational events from Roche.

### Open Research Badges

This article has earned an Open Data badge for making publicly available the digitally‐shareable data necessary to reproduce the reported results. The data is available in the Supporting Information section.

This article has earned an open data badge “**Reproducible Research**” for making publicly available the code necessary to reproduce the reported results. The results reported in this article could fully be reproduced.

## Supporting information

Supporting Information

## Data Availability

The data that support the findings of this study are available in the Supporting Information of this article.

## Appendix A Deriving Estimating Equations for α and β

Write $o_{i}\left({\hat{\gamma }}_{i}\right)=\left(\rho_{i}\sigma_{i}/\delta_{i}\right)\left({\hat{\gamma }}_{i}-\gamma_{i}\right)$ and $V_{i}^{2}=v_{i}^{2}+\tau^{2}$ in model (6), so that

(A1)
$${\hat{\theta }}_{i}|{\hat{\gamma }}_{i}∼N\left(\alpha +\beta \gamma_{i}+o_{i}\left({\hat{\gamma }}_{i}\right),V_{i}^{2}\right).$$

The marginal model ${\hat{\gamma }}_{i}∼N\left(\gamma_{i},\delta_{i}^{2}\right)$ does not depend on α or β. When differentiating the log‐likelihood function with respect to either of these parameters, only terms from the n conditional densities ${\hat{\theta }}_{i}|{\hat{\gamma }}_{i}$ contribute. The maximum likelihood estimates of α and β therefore satisfy the maximum likelihood estimating equations from model (A1), where other parameters are replaced by their maximum likelihood estimates. Hence, if we define ${\hat{o}}_{i}\left({\hat{\gamma }}_{i}\right)=\left(\rho_{i}\sigma_{i}/\delta_{i}\right)\left({\hat{\gamma }}_{i}-{\hat{\gamma }}_{i,MLE}\right)$, ${\hat{\theta }}_{i}^{†}={\hat{\theta }}_{i}-{\hat{o}}_{i}\left({\hat{\gamma }}_{i}\right)$, $w_{i}={\hat{V}}_{i}^{-2}$, ${\bar{\hat{\theta }}}^{†}$ and ${\bar{\gamma }}_{MLE}$ to be the *weighted by* $w_{i}$ mean of the ${{\hat{\theta }}^{†}}_{i}$ and ${\hat{\gamma }}_{i,MLE}$, respectively, the maximum likelihood estimates of α and β satisfy the estimating equations

(A2)
$$\hat{\beta }=\frac{\sum_{i=1}^{n}w_{i}\left({{\hat{\theta }}^{†}}_{i}-{\bar{\hat{\theta }}}^{†}\right)\left(\gamma_{i,MLE}-{\bar{\gamma }}_{MLE}\right)}{\sum_{i=1}^{n}w_{i}{\left(\gamma_{i,MLE}-{\bar{\gamma }}_{MLE}\right)}^{2}}$$

and

(A3)
$$\hat{\alpha }={\bar{\hat{\theta }}}^{†}-\hat{\beta }{\bar{\gamma }}_{MLE}.$$

Estimating Equations (A2) and (A3) can be used to estimate α and β, similar to the way that estimating Equation (5) can be used to estimate ${\hat{\gamma }}_{i,MLE}$.

## Appendix B A Mathematical Result Required to Derive a Bias Adjusted Estimating Equation for $\tau^{2}$

As explained in Section 4, we treat $\hat{\alpha }$, $\hat{\beta ,}$ and ${\hat{\tau }}^{2}$ as fixed constants but this is not the case for the ${\hat{\gamma }}_{i,MLE}$. Let $w_{1i}$ and $w_{2i}$ be the first and second entries of the row vector ${\left(C_{\left(\hat{\beta },1\right)}^{T}{\hat{W}}_{i}C_{\left(\hat{\beta },1\right)}\right)}^{-1}C_{\left(\hat{\beta },1\right)}^{T}{\hat{W}}_{i}$, respectively. Then, from estimating Equation (5), we have

(B1)
$${\hat{\gamma }}_{i,MLE}=w_{1i}{\hat{\theta }}_{i}^{*}+w_{2i}{\hat{\gamma }}_{i}=w_{1i}\left({\hat{\theta }}_{i}-\hat{\alpha }\right)+w_{2i}{\hat{\gamma }}_{i}$$

where in estimating Equation (9) we evaluate

(B2)
$${\hat{\mu }}_{i}\left({\hat{\gamma }}_{i}\right)=\hat{\alpha }+\hat{\beta }{\hat{\gamma }}_{i,MLE}+\left(\rho_{i}\sigma_{i}/\delta_{i}\right)\left({\hat{\gamma }}_{i}-{\hat{\gamma }}_{i,MLE}\right).$$

Substituting Equation (B1) into Equation (B2), and some algebra, gives

(B3)
$${\hat{\mu }}_{i}\left({\hat{\gamma }}_{i}\right)=a_{i}+b_{i}{\hat{\theta }}_{i}+c_{i}{\hat{\gamma }}_{i},$$

where $a_{i}=\hat{\alpha }\left(1-\left(\hat{\beta }-\rho_{i}\sigma_{i}/\delta_{i}\right)w_{1i}\right)$, $b_{i}=\left(\hat{\beta }-\rho_{i}\sigma_{i}/\delta_{i}\right)w_{1i}$, $c_{i}=\left(\hat{\beta }-\rho_{i}\sigma_{i}/\delta_{i}\right)w_{2i}+\rho_{i}\sigma_{i}/\delta_{i}$. All $a_{i}$, $b_{i}$, and $c_{i}$ are treated as fixed constants, because $\hat{\alpha }$, $\hat{\beta ,}$ and ${\hat{\tau }}^{2}$ are treated as such.

The term ${\hat{\mu }}_{i}\left({\hat{\gamma }}_{i}\right)=a_{i}+b_{i}{\hat{\theta }}_{i}+c_{i}{\hat{\gamma }}_{i}$ is an estimate of the conditional mean of ${\hat{\theta }}_{i}|{\hat{\gamma }}_{i}$. We assume this estimate is unbiased, so that $E(\left({\hat{\theta }}_{i}-a_{i}-b_{i}{\hat{\theta }}_{i}-c_{i}{\hat{\gamma }}_{i}\right)|{\hat{\gamma }}_{i})=0$. Hence

$$\begin{array}{ccc} E({\left({\hat{\theta }}_{i}-{\hat{\mu }}_{i}\left({\hat{\gamma }}_{i}\right)\right)}^{2}|{\hat{\gamma }}_{i}) & = & Var\left(\left(1-b_{i}\right){\hat{\theta }}_{i}|{\hat{\gamma }}_{i}\right)={\left(1-b_{i}\right)}^{2}Var\left({\hat{\theta }}_{i}|{\hat{\gamma }}_{i}\right)\\ & = & {\left(1-b_{i}\right)}^{2}\left(v_{i}^{2}+\tau^{2}\right), \end{array}$$

so that

(B4)
$${\left(1-b_{i}\right)}^{-2}E\left({\left({\hat{\theta }}_{i}-{\hat{\mu }}_{i}\left({\hat{\gamma }}_{i}\right)\right)}^{2}|{\hat{\gamma }}_{i}\right)=v_{i}^{2}+\tau^{2}.$$

Equation (B4) is the key mathematical result required to motivate our proposed bias adjusted estimator.
