# Supplementary material for: A New Frequentist Implementation of the Daniels and Hughes Bivariate Meta‐Analysis Model for Surrogate Endpoint Evaluation
Source: Biom J. 2025 Mar 19;67(2):e70048. doi: 10.1002/bimj.70048 (PMC11921291; doi:10.1002/bimj.70048)
Supplement: Supplementary file 1 — Supporting Information [file BIMJ-67-e70048-s001.zip › Supplementary_Codes/Generate_ss_supplement.pdf]

A new frequentist implementation of the Daniels and Hughes bivariate meta-analysis model for surrogate endpoint evaluation: generating simulation study results

Dan Jackson et al

2025-01-03

Load packages

These codes require several R packages and we begin by loading them.

```
##    name          version                module
## 62 JAGS 4.3.1-foss-2021a JAGS/4.3.1-foss-2021a
```

Write a function to take 5 vectors (the 2 sets of estimates and their within-study variances and correlations) and make data suitable for analysis

We now write a function to create data suitable for analysis. This is because it will often be easiest for the analyst to provide the estimates for the outcome, the surrogate, and the corresponding within-study variances and correlations. The function below creates a dataframe with the estimates in the required format and a list containing the within-study covariance matrices.

```
# write function
data.manipulate<-function(out.est, resp.est, out.var, resp.var, rho)
{
# Form a dataframe where each ordered pair of estimates appears for each
# study in turn. For each study the outcome appears first and the response
# appears second. We want to know if the response (second variable)
# is a good surrogate for the outcome, so we "regress" the outcome (first
# variable) on the response (second).
N<-length(resp.est)
odd.numbers<-seq(from=1, length.out=N, by=2)
Y<-rep(NA, 2*N)
Y[odd.numbers]<-out.est
Y[odd.numbers+1]<-resp.est
# Now create the dataframe
my.data<-data.frame(study=rep(1:N, each=2), var=rep(c("outcome", "response"), N), yi=Y)
# Form the within-study variance matrix V for all studies.
V_list<-list()
# Now create the within-study covariance matrices
for(i in 1:N)
{
V_list[[i]]<-matrix(nrow=2, ncol=2)
V_list[[i]][1,1]<-out.var[i]
V_list[[i]][2,2]<-resp.var[i]
V_list[[i]][2,1]<-rho[i]*((resp.var[i]*out.var[i])^0.5)
V_list[[i]][1,2]<-V_list[[i]][2,1]
}
# Return the dataframe and the list of covariance matrices
return(list(my.data=my.data, V_list=V_list))
}
```

Write the optimisation function to be maximised

We now write a function that evaluates (minus) the log likelihood given the study estimates (in the order created by the previous data.manipulate function) and their within-study covariance matrix. This covariance matrix is easily calculated as the bldiag of the list of covariance matrix also created by this function.

The function allows tau2 (tau-squared) to be fixed (tau2 argument specified to be a constant) or estimated (the default, tau2=NA) when computing the log likelihood.

```
optfun <- function(par, yi, V, tau2=NA) {
  terms  <- ifelse(is.na(tau2),  3, 2)
  # Set alpha and beta as first 2 parameters
  alpha <- par[1]
  beta  <- par[2]
  # Unless tau2 is fixed log tau2 is set as third parameter
  tau2  <- ifelse(is.na(tau2),  exp(par[3]), tau2)
  # Set the gammas as other parameters
  gammai <- par[-c(1:terms)]
  # Generate the mean using "Wolfgang's trick"
  mean.o <- alpha + beta * gammai
  mean.r <- gammai
  mean   <- c(rbind(mean.o, mean.r))
  # Set the variance structure as total variance
  diag(V) <- diag(V) + c(tau2, 0)
  # Compute minus log likelihood
  lli <- dmvnorm(yi, mean=mean, sigma=V, log=TRUE)
  ll <- -1*lli
  return(ll)
}
```

Write a function to perform maximum likelihood estimation

We now write a function to compute the maximum likelihood estimates for a given dataset, where the dataset is created by the data.manipulate function. We must also specify the optimisation method (BFGS works well) and specify tau2 if it is held fixed. M and rel relate to the optimisation tolerances and can be changed.

```
do_estimation<-function(my.data, V_list, opt_method, tau2=NA,
                        M=100000, rel=0.0000000000000001)
{
# Create the within-study variance matrix for all study estimates
  V<-bldiag(V_list)
# Create starting values for the optimiser.
# Start at 0 for alpha and beta and log(0.5) for log(tau2)
# These could be changed or made arguments of the function
# We use the study estimates of the gamma_i as the initial values
  ifelse(is.na(tau2), start <- c(0,0, log(.5),my.data$yi[my.data$var == "response"]),
         start <- c(0,0,my.data$yi[my.data$var == "response"]))
# Now use optim to compute the MLEs
  res<-optim(start, optfun, yi=my.data$yi, V=V, tau2=tau2,
             control = list(maxit = M, reltol=rel),
             method=opt_method)
 # Get the Hessian
  H <- hessian(optfun, res$par, yi=my.data$yi, V=V,
               tau2=tau2)
  Vpar <- ginv(H) # Use ginv to avoid problems if hat tau2=0, as explained in the paper
  se <- sqrt(diag(Vpar))
  # Return estimates, standard errors, convergence code and optimum likelihood
  return(list(est=res$par, se=se, conv=res$convergence, best=res$value))
}
```

Write a function to check maximum likelihood estimates have been obtained and produce the bias adjusted estimate of tau2

We now write a function to check the maximum likelihood estimates have been obtained and compute the bias adjustes estimate. This function requires the data in the usual format and also MLE, an object created by the previous do estimation function.

```
check_adjust<-function(my.data, V_list, MLE, tau2=NA)
{
V<-bldiag(V_list)
N<-nrow(my.data)/2
# "Reverse engineer" the within study correlations
# We formed within-study covariance matrices from these correlations
# and the corresponding variances when creating datsets
# but now we require the correlations again
# again.
rho<-NULL
for(i in 1:N)
{
rho[i]<- V_list[[i]][1,2]/(sqrt(V_list[[i]][1,1] * V_list[[i]][2,2]))
}
# Extract all the maximum likelihood estimates
  alpha_hat<-MLE$est[1]; beta_hat<-MLE$est[2];
  if(is.na(tau2)){tau2_hat<-exp(MLE$est[3]); gamma_i_hat<-MLE$est[-(1:3)]}
  if(is.na(tau2)==FALSE){tau2_hat<-tau2; gamma_i_hat<-MLE$est[-(1:2)]}
  ################################################################################
  # now check the estimation using the estimating equations in paper.
  # compute some values that will be useful later
  # N<-nrow(my.data)/2
  # Extract the within study variances from the covariance matrix
  odd.numbers<-seq(from=1, length.out=N, by=2); even.numbers<-odd.numbers+1
  out.var<-(diag(V))[odd.numbers]; resp.var<-(diag(V))[even.numbers]
  v_var<-out.var*(1-rho^2)
  ################################################################################
  # check gamma_i MLES and compute adjustment to tau-sq
  # estimation terms - latter only needed if tau^2 estimated
  gamma_check<-NULL;  bi<-NULL
  # Compute the theta_hat_star as in the paper
  outcome_ests_star<-(my.data$yi[odd.numbers] - alpha_hat)
  for (i in 1:N)
  {
  diag(V_list[[i]])<-diag(V_list[[i]]) + c(tau2_hat, 0)
  W_mat<-solve(V_list[[i]])
  C_vector<-matrix(c(beta_hat, 1), nrow=2, ncol=1)
  outcome_i<-matrix(c(outcome_ests_star[i], my.data$yi[even.numbers][i]), ncol=1)
  # Now compute gamma_i_hat from estimating equations
  # The gamma_check from the estimating equation will be the
  # same as the gamma_i_hat from numerical optimisation if all is well
  gamma_check[i]<-as.numeric(1/(t(C_vector)%*%W_mat%*%C_vector)*
                             (t(C_vector))%*%W_mat%*%outcome_i)
  # Also compute the bias correction terms b_i
  # Using numerical methods without algebraic simplification
  w_i<-c(as.numeric(1/(t(C_vector)%*%W_mat%*%C_vector))*(t(C_vector))%*%W_mat)
  bi[i]<-w_i[1]*(beta_hat-rho[i] *sqrt(out.var[i]/resp.var[i]))
```

```
}
#################################################################################
# Now that the gamma_i have been "estimated" using estimating equations
# We can now check tau2, and compute its bias adjusted estimate
# These tau2 outputs will be NA unless tau2 is estimated
tau2_check<-NA; tau2_correction<-NA; tau2_correction_algebra<-NA
# If tau-sq is estimated then check the estimation using estimating equation
# and compute the bias corrected version
if(is.na(tau2)==TRUE)
{
var_est_sq<-(v_var + tau2_hat)^2
cond_mean<-alpha_hat+beta_hat*gamma_i_hat +
  rho *sqrt(out.var/resp.var)*(my.data$yi[even.numbers] - gamma_i_hat)
tau2_check<-max(sum(((my.data$yi[odd.numbers] - cond_mean)^2 - v_var)/
                      var_est_sq)/sum(1/var_est_sq),0)

# now compute adjusted tau2_hat - the numerical way
dof_correction<-1/((1-bi)^2)
tau2_correction<-sum(((dof_correction*(my.data$yi[odd.numbers] -
                                         cond_mean)^2) -
                        v_var)/var_est_sq)/sum(1/var_est_sq)
tau2_correction<-max(0, tau2_correction)


# Now compute adjusted tau2 the algebraic/clean way
# Where much of the computation of the adjustment terms
# b_i is done mathematically.
# Use both numerical and algebraic/clean way to confirm
# numerical methods for the b_i have worked.
# Formula for b_i in the paper follows as bi_algebra
top<-(beta_hat*sqrt(resp.var) - rho*sqrt(out.var))^2
bot<-top + v_var + tau2_hat
bi_algebra<-top/bot

dof_correction_algebra<-1/((1-bi_algebra)^2)
tau2_correction_algebra<-sum(((dof_correction_algebra*(my.data$yi[odd.numbers] -
                                                         cond_mean)^2) -
                                v_var)/var_est_sq)/sum(1/var_est_sq)
tau2_correction_algebra<-max(0, tau2_correction_algebra)

}
#################################################################################
# Finally check alpha and beta MLES
# Using their estimating equations
weights<-1/(v_var + tau2_hat)
offset<-rho *sqrt(out.var/resp.var)*(my.data$yi[odd.numbers+1] - gamma_i_hat)
theta_hat_dagger<-my.data$yi[odd.numbers] - offset
theta_hat_dagger_mean<-sum(weights*theta_hat_dagger)/sum(weights)
gamma_hat_mean<-sum(weights*gamma_i_hat)/sum(weights)
beta_hat_check<-sum(weights* (theta_hat_dagger-theta_hat_dagger_mean)*
                      (gamma_i_hat-gamma_hat_mean))/
  sum(weights*(gamma_i_hat-gamma_hat_mean)^2)
alpha_hat_check<-theta_hat_dagger_mean - beta_hat_check * gamma_hat_mean
```

```r
  ################################################################################
  # All work now done. Return all "check" - parameters re-estimated using their estimating
  # equations to check MLE contains MLEs and also the bias corrected tau2
  # where the b_i were computed numerically: tau2_correction
  # and using algebraic simplification: tau2_correction_algebra
  # We therefore have lots of numerical checks and the bias corrected estimate of tau2
  # by using this function.
return(list(tau2_hat_check=tau2_check, tau2_correction=tau2_correction,
            tau2_correction_algebra=tau2_correction_algebra,
            alpha_hat_check=alpha_hat_check, beta_hat_check = beta_hat_check,
            gamma_hat_check=gamma_check))

}
```

Now write a function to do all estimation, checking and bias correction

We have several different functions that perform different roles. We now write a function that will make use of previous functions to conveniently perform all frequentist analysis. It requires the data and within-study covariance matrices in the usual form plus details relating to the optimisation method used.

```
do_estimation_full<-function(my.data, V_list, opt_method,
                             M=100000, rel=0.0000000000000001)
{
# First do standard MLE estimation
MLE_estimation<-do_estimation(my.data, V_list, opt_method, tau2=NA, M=M, rel=rel)
# Now check MLE estimation satisfies estimating equations, so the optimisation worked.
# The next function also produces adjusted tau2 estimate because tau2=NA
# in the previous do_estimation call
checking_adjustment<-check_adjust(my.data, V_list, MLE_estimation, tau2=NA)
# Extract the adjusted tau2 estimate - using algebraic correction terms
# (could use numerical version also)
tau2_correction_algebra<-checking_adjustment$tau2_correction_algebra
# Now do the adjusted estimation
# By constraining tau2 to be the adjusted estimate
MLE_estimation_fixed<-do_estimation(my.data, V_list, opt_method,
                                    tau2=tau2_correction_algebra,
                                    M=M, rel=rel)
# Check adjusted MLEs satisfy estimating equations
checking_adjustment_fixed<-check_adjust(my.data, V_list, MLE_estimation_fixed,
                                        tau2=tau2_correction_algebra)
# All work now done.
# Store the results - put all the above together
# First extract the estimates of alpha, both in MLE analysis and adjusted
results<-data.frame(alpha=c(MLE_estimation$est[1], MLE_estimation_fixed$est[1]))
row.names(results)<-c("MLE", "Adjusted")
# Now add the checks for alpha
# that should be the same as the alpha estimates
results$alpha_check<-c(checking_adjustment$alpha_hat_check,
                       checking_adjustment_fixed$alpha_hat_check)
# Add the standard errors
results$alpha_se<-c(MLE_estimation$se[1], MLE_estimation_fixed$se[1])

# Now do the same for beta
results$beta<-c(MLE_estimation$est[2], MLE_estimation_fixed$est[2])
results$beta_check<-c(checking_adjustment$beta_hat_check,
                      checking_adjustment_fixed$beta_hat_check)
results$beta_se<-c(MLE_estimation$se[2], MLE_estimation_fixed$se[2])

# Now get the tau2 estimates
results$tau2<-c(exp(MLE_estimation$est[3]), tau2_correction_algebra)
# now get tau2 checks
# for the standard MLE analysis we use the estimating equation
# for the adjusted analysis we instead take the messy/ non algebraic
# adjusted estimate - this is a different type of check that all is well
results$tau2_check<-c(checking_adjustment$tau2_hat_check,
                       checking_adjustment$tau2_correction)
# For the gammas, just report the maximum absolute difference between
```

```
# numerical estimates and RHS of estimating equations, for brevity.
# If this is small then all is well
results$gamma_check<-c(max(abs(checking_adjustment$gamma_hat_check -
                                 MLE_estimation$est[-c(1:3)])),
                       max(abs(checking_adjustment_fixed$gamma_hat_check -
                                 MLE_estimation_fixed$est[-c(1:2)])))
# Finally return all the results
results
}
```

Write a function to do Bayesian analysis

It is also useful to have a Bayesian version of the analysis to compare frequentist results to (see the main paper for full details).

```
bayes_est<-function(my.data, V_list, tau2, HDR=FALSE)
{
# Data required in the usual way.
# Note tau2 is just used to create starting values for the MCMC chains
# is not needed otherwise!
N<-length(V_list)
Y<-matrix(nrow=N, ncol=2)
# Extract data for jags.
Y[,2]<-my.data$yi[my.data$var=="outcome"]
Y[,1]<-my.data$yi[my.data$var=="response"]
Var_response<-rep(NA, N); Var_outcome<-rep(NA, N); Cov<-rep(NA, N)
for(i in 1:N)
{
Var_response[i]<-V_list[[i]][2,2]
Var_outcome[i]<-V_list[[i]][1,1]
Cov[i]<-V_list[[i]][2,1]
}
# Now run the Bayesian model
model.file<-"NICE_tech_DSU.txt"
## For reproducibility, need to add random seed to each initiated chain
inits<-list(list(psi.2=tau2, .RNG.name="base::Wichmann-Hill", .RNG.seed=17334734),
            list(psi.2=tau2/2, .RNG.name="base::Wichmann-Hill", .RNG.seed=2121261))

data.bugs<-list()
data.bugs$Y<-Y  ## y[1] is response/surrogate, y[2] is outcome
data.bugs$N<-N  ## Number of studies
data.bugs$Var_response<-Var_response
data.bugs$Var_outcome<-Var_outcome
data.bugs$Cov<-Cov


jagsobj<-jags.model(model.file,data=data.bugs,n.chains=2,quiet=TRUE,inits=inits)


update(jagsobj,n.iter=1000) ## Burn-in iterations to settle MCMC chain
results <- coda.samples(jagsobj, c("lambda0", "lambda1", "psi2.sq"),
                        n.iter=10000, thin=2)
# Could also use model diagnostics
# diagnostics<-heidel.diag(results)
# Produce a table of results
results_summary<-summary(results)
answers<-cbind(results_summary$statistics, results_summary$quantiles)[, c(1, 7, 5 ,9)]
colnames(answers)<-c("mean", "median", "lower", "upper")
if (HDR==TRUE)
{
#############################################################
######### Extra lines of code for HDR ######################
######### Only implemented for examples ####################
######### First put the two chains together ################
```

```
combine.samples<-structure(c(results[[1]][,1], results[[2]][,1],
                             results[[1]][,2], results[[2]][,2],
                             results[[1]][,3], results[[2]][,3]),
                           .Dim = c(10000L, 3L),
                           .Dimnames = list(NULL, c("lambda0", "lambda1", "psi2.sq")),
                           class="mcmc")
######## Next get HPD CI ####################################
HDR<-as.data.frame(HPDinterval(combine.samples, prob = 0.95))
######## Add HPD CI to the Bayesian results #################
colnames(HDR)<-c("HDR_lower", "HDR_upper")
answers<-cbind(answers, HDR)
######## End of extra lines of code to add HDR ##############
#############################################################
}
answers
}
```

Write a function to simulate one dataset

```
# Set the seed for reproducibility
set.seed(123456)
# Write a function to simulate the data
# We require the within-study variances (out.var and resp.var)
# and the within-study correlations
# Defaults are alpha=1, beta=2, tau2=0.5
# The length of the vectors determined the number of studies
sim_data<-function(out.var, resp.var, rho, alpha=1, beta=2, tau2=0.5)
{
  ##################################################################
  ## simulate data ################################################
  N<-length(out.var)
  # simulate the gammas - U(-5,5)
  gammas<-runif(N, min=-5, max=5)
  # generate the means for both types of outcomes
  mean.o <- alpha + beta * gammas
  mean.r <- gammas
  # Use Wolfgang's trick to form mean for both outcomes
  the_mean   <- c(rbind(mean.o, mean.r))
  # generate within-study variance matrices
  V<-list()
  for(i in 1:N)
  {
    V[[i]]<-matrix(nrow=2, ncol=2)
    V[[i]][1,1]<-out.var[i]
    V[[i]][2,2]<-resp.var[i]
    V[[i]][2,1]<-rho[i]*((resp.var[i]*out.var[i])^0.5)
    V[[i]][1,2]<-V[[i]][2,1]
  }
  V_list<-V; V_list_fix<-V
  # Form matrix of within-study variances
  V<-bldiag(V)
  # Compute total variance matrix
  Total_var<-V
  diag(Total_var)<-diag(Total_var) + c(tau2, 0)
  # Simulate data
  Y<-rmvnorm(1, mean=the_mean, sigma=Total_var)
  # Return dataframe and within study variance matrix
  odd.numbers<-seq(from=1, length.out=N, by=2)
  #################################################################################
  ### Put data in form for model fitting
  model.fitting.data<-data.manipulate(Y[odd.numbers], Y[odd.numbers+1], out.var, resp.var, rho)
model.fitting.data
  }
```

Write a function to do a simulation study

This function simply calls the previous function to simulate data many times and uses the model fitting functions to produce results.

```
simulation_study<-function(out.var, resp.var, rho, alpha=1, beta=2, tau2=0.5, sims=50)
{
# sims is the number of simulations to run - 1000 needed for the paper
listNA<-rep(NA, sims)
# Store MLE results
frequentist_MLE<-data.frame(alpha=listNA, alpha_check=listNA,
                            alpha_se=listNA,
                            beta=listNA, beta_check=listNA,
                            beta_se=listNA, tau2=listNA,
                            tau2_check=listNA,  gamma_check=listNA)
# Store adjusted results
frequentist_adjusted<-frequentist_MLE
# Store Bayesian results
bayesian_posterior<-data.frame(alpha = listNA, median_alpha=listNA,
                               lower_alpha=listNA, upper_alpha=listNA,
                               beta = listNA, median_beta=listNA,
                               lower_beta=listNA, upper_beta=listNA,
                               tau2 = listNA, median_tau2=listNA,
                               lower_tau2=listNA, upper_tau2=listNA)

# Now do everything "sims times", eg sims=1000 times
for(i in 1:sims)
{
# Simulate data set
simulation_data<-sim_data(out.var, resp.var, rho, alpha, beta, tau2)
# Do all frequentist estimation
frequentist<-do_estimation_full(simulation_data$my.data,
                                       simulation_data$V_list, "BFGS")

# Extract MLE and adjusted results
frequentist_MLE[i,]<-frequentist[1,]
frequentist_adjusted[i,]<-frequentist[2,]
# Extract Bayesian results 0 make sure we do not start at 0 with max tau2, 0.1
# The last argument is the starting value for the MCMC, which does not like starting at 0
bayesian<-bayes_est(simulation_data$my.data, simulation_data$V_list, max(tau2, 0.1))
bayesian_posterior[i,]<-c(t(bayesian))

}
# Put all results together in one big data frame and return them
colnames(frequentist_adjusted)<-paste("Adj", colnames(frequentist_adjusted), sep="_")
colnames(bayesian_posterior)<-paste("Bayes", colnames(bayesian_posterior), sep="_")

results<-cbind(frequentist_MLE, frequentist_adjusted, bayesian_posterior)
# Store a few other true parameter values
# The average correlation will be the common correlation when this
# is the same across studies.
# Useful to have these values for reference later.
results$true_alpha<-alpha
results$true_beta<-beta
results$true_tau2<-tau2
```

```
results$corr<-mean(rho)
results$N<-length(out.var)
# Output the results
results
}
```

Now run the simulation study required

```r
# Set up all 54 combinations of parameters
corrs<-c(rep(0, 18), rep(0.4, 18), rep(0.8, 18))
corrs; length(corrs)
```

```
##  [1] 0.0 0.0 0.0 0.0 0.0 0.0 0.0 0.0 0.0 0.0 0.0 0.0 0.0 0.0 0.0 0.0 0.0 0.0 0.4
## [20] 0.4 0.4 0.4 0.4 0.4 0.4 0.4 0.4 0.4 0.4 0.4 0.4 0.4 0.4 0.4 0.4 0.8 0.8
## [39] 0.8 0.8 0.8 0.8 0.8 0.8 0.8 0.8 0.8 0.8 0.8 0.8 0.8 0.8 0.8 0.8
```

```
## [1] 54
```

```r
alphas<-c(rep(0, 9), rep(0.25, 9), rep(0, 9), rep(0.25, 9), rep(0, 9), rep(0.25, 9))
alphas; length(alphas)
```

```
##  [1] 0.00 0.00 0.00 0.00 0.00 0.00 0.00 0.00 0.00 0.25 0.25 0.25 0.25 0.25 0.25
## [16] 0.25 0.25 0.25 0.00 0.00 0.00 0.00 0.00 0.00 0.00 0.00 0.00 0.25 0.25 0.25
## [31] 0.25 0.25 0.25 0.25 0.25 0.25 0.00 0.00 0.00 0.00 0.00 0.00 0.00 0.00 0.00
## [46] 0.25 0.25 0.25 0.25 0.25 0.25 0.25 0.25 0.25
```

```
## [1] 54
```

```r
betas<-rep(c(rep(0, 3), rep(0.4, 3), rep(0.8, 3)),6)
betas; length(betas)
```

```
##  [1] 0.0 0.0 0.0 0.4 0.4 0.4 0.8 0.8 0.8 0.0 0.0 0.0 0.4 0.4 0.4 0.8 0.8 0.8 0.0
## [20] 0.0 0.0 0.4 0.4 0.4 0.8 0.8 0.8 0.0 0.0 0.0 0.4 0.4 0.4 0.8 0.8 0.8 0.0 0.0
## [39] 0.0 0.4 0.4 0.4 0.8 0.8 0.8 0.0 0.0 0.0 0.4 0.4 0.4 0.8 0.8 0.8
```

```
## [1] 54
```

```r
tau2s<-rep(rep(c(0, 0.5, 1), 18))
tau2s; length(tau2s)
```

```
##  [1] 0.0 0.5 1.0 0.0 0.5 1.0 0.0 0.5 1.0 0.0 0.5 1.0 0.0 0.5 1.0 0.0 0.5 1.0 0.0
## [20] 0.5 1.0 0.0 0.5 1.0 0.0 0.5 1.0 0.0 0.5 1.0 0.0 0.5 1.0 0.0 0.5 1.0 0.0 0.5
## [39] 1.0 0.0 0.5 1.0 0.0 0.5 1.0 0.0 0.5 1.0 0.0 0.5 1.0 0.0 0.5 1.0
```

```
## [1] 54
```

```r
# Set number of simulations
n_sim<-1000
# Produce results and write spreadsheets with the results
# These results will be processed later on.
for(i in 1:54)
{
results<-simulation_study((1:20)/20, (1:20)/20, rep(corrs[i], 20),
                 alpha=alphas[i], beta=betas[i], tau2=tau2s[i], sims=n_sim)
name<-paste("results", sep="_", i, ".csv")
write.csv(results, name)
}
```

```r
sessionInfo()
```

```
## R version 4.3.1 (2023-06-16)
## Platform: x86_64-pc-linux-gnu (64-bit)
## Running under: Rocky Linux 8.6 (Green Obsidian)
##
## Matrix products: default
## BLAS/LAPACK: FlexiBLAS OPENBLAS;  LAPACK version 3.9.0
##
## locale:
##  [1] LC_CTYPE=en_US.UTF-8       LC_NUMERIC=C
##  [3] LC_TIME=en_US.UTF-8        LC_COLLATE=en_US.UTF-8
##  [5] LC_MONETARY=en_US.UTF-8    LC_MESSAGES=C
##  [7] LC_PAPER=en_US.UTF-8       LC_NAME=C
##  [9] LC_ADDRESS=C               LC_TELEPHONE=C
## [11] LC_MEASUREMENT=en_US.UTF-8 LC_IDENTIFICATION=C
##
## time zone: :/etc/localtime
## tzcode source: system (glibc)
##
## attached base packages:
## [1] stats     graphics  grDevices utils     datasets  methods   base
##
## other attached packages:
##  [1] rjags_4-16          coda_0.19-4.1       azcore_1.0.0
##  [4] ggplot2_3.5.1       MASS_7.3-60         mvtnorm_1.3-1
##  [7] metafor_4.6-0       numDeriv_2016.8-1.1 metadat_1.2-0
## [10] Matrix_1.6-3
##
## loaded via a namespace (and not attached):
##  [1] vctrs_0.6.4       nlme_3.1-163      cli_3.6.3         knitr_1.49
##  [5] rlang_1.1.4       xfun_0.49         generics_0.1.3    glue_1.8.0
##  [9] colorspace_2.1-0  htmltools_0.5.8.1 fansi_1.0.5       scales_1.3.0
## [13] rmarkdown_2.29    grid_4.3.1        tibble_3.2.1      evaluate_0.23
## [17] munsell_0.5.0     fastmap_1.2.0     yaml_2.3.10       lifecycle_1.0.4
## [21] compiler_4.3.1    dplyr_1.1.4       mathjaxr_1.6-0    pkgconfig_2.0.3
## [25] rstudioapi_0.15.0 lattice_0.22-5    digest_0.6.33     R6_2.5.1
## [29] tidyselect_1.2.1  utf8_1.2.4        pillar_1.9.0      magrittr_2.0.3
## [33] withr_2.5.2       tools_4.3.1       gtable_0.3.4
```
