# Supplementary material for: A New Frequentist Implementation of the Daniels and Hughes Bivariate Meta‐Analysis Model for Surrogate Endpoint Evaluation
Source: Biom J. 2025 Mar 19;67(2):e70048. doi: 10.1002/bimj.70048 (PMC11921291; doi:10.1002/bimj.70048)
Supplement: Supplementary file 1 — Supporting Information [file BIMJ-67-e70048-s001.zip › Supplementary_Codes/SIM_STUDY_RESULTS_sessionInfo.html]

A new frequentist implementation of the Daniels and Hughes bivariate meta-analysis model for surrogate endpoint evaluation: Simulation study results


# A new frequentist implementation of the Daniels and Hughes bivariate meta-analysis model for surrogate endpoint evaluation: Simulation study results

#### Dan Jackson

#### 2025-01-03

# Simulation study design (see also the main paper)

We used \(n=20\) studies throughout.
We assumed that the 20 values of \(\sigma\_i^2=
\delta\_i^2=0.05, 0.1, ...1.00\) are fixed for each simulated
dataset. We took \(\sigma\_i^2=
\delta\_i^2\) for all studies, so that the criterion \(\beta=\rho\_i\sigma\_i/\delta\_i\) is simply
\(\beta=\rho\_i\). We varied four
parameters, where in total 54 combinations of parameter values were
explored. 1000 simulations where used in each setting. See the main
paper for more details, but briefly we used:

1. Three different values of \(\rho = 0,
   0.4, 0.8\).
2. Three different values of \(\beta = 0,
   0.4, 0.8\).
3. Three different values of \(\tau^2=0,
   0.5, 1\).
4. Two values of \(\alpha=0,
   0.25\).

Three analysis methods were used: 1) standard maximum likelihood, 2)
the proposed bias adjusted method, 3) A Bayesian analysis. Again see the
main paper for full details.

We present results across all 54 scenarios and also for the 18
settings where \(\beta=\rho\_i\) (and
maximum likelihood estimation of \(\tau^2\) should perform adequately).

Eight numerical checks are made, these are the maximum absolute
differences between estimates obtained numerically (\(\alpha\_i\), \(\beta\), \(\tau^2\), \(\gamma\_i\); maximum likelihood estimates
and adjusted estimates) and estimates obtained from the estimating
equations. In all cases these differences are very small and so we have
agreement to within numerical error.

# Bias for alpha

# Bias for beta

# Bias for tau2

# Coverage for alpha

# Coverage for beta

# Comparing posterior means and medians of tau2

```
## R version 4.3.1 (2023-06-16)
## Platform: x86_64-pc-linux-gnu (64-bit)
## Running under: Rocky Linux 8.6 (Green Obsidian)
## 
## Matrix products: default
## BLAS/LAPACK: FlexiBLAS OPENBLAS;  LAPACK version 3.9.0
## 
## locale:
##  [1] LC_CTYPE=en_US.UTF-8       LC_NUMERIC=C              
##  [3] LC_TIME=en_US.UTF-8        LC_COLLATE=en_US.UTF-8    
##  [5] LC_MONETARY=en_US.UTF-8    LC_MESSAGES=C             
##  [7] LC_PAPER=en_US.UTF-8       LC_NAME=C                 
##  [9] LC_ADDRESS=C               LC_TELEPHONE=C            
## [11] LC_MEASUREMENT=en_US.UTF-8 LC_IDENTIFICATION=C       
## 
## time zone: :/etc/localtime
## tzcode source: system (glibc)
## 
## attached base packages:
## [1] stats     graphics  grDevices utils     datasets  methods   base     
## 
## other attached packages:
##  [1] lubridate_1.9.3 forcats_1.0.0   stringr_1.5.1   dplyr_1.1.4    
##  [5] purrr_1.0.2     readr_2.1.4     tidyr_1.3.0     tibble_3.2.1   
##  [9] ggplot2_3.5.1   tidyverse_2.0.0 rsimsum_0.13.0 
## 
## loaded via a namespace (and not attached):
##  [1] gtable_0.3.4      jsonlite_1.8.7    compiler_4.3.1    tidyselect_1.2.1 
##  [5] jquerylib_0.1.4   textshaping_0.3.7 systemfonts_1.0.5 scales_1.3.0     
##  [9] yaml_2.3.10       fastmap_1.2.0     R6_2.5.1          labeling_0.4.3   
## [13] generics_0.1.3    knitr_1.49        backports_1.4.1   checkmate_2.3.2  
## [17] munsell_0.5.0     tzdb_0.4.0        bslib_0.6.0       pillar_1.9.0     
## [21] rlang_1.1.4       utf8_1.2.4        stringi_1.8.2     cachem_1.1.0     
## [25] xfun_0.49         sass_0.4.9        timechange_0.2.0  cli_3.6.3        
## [29] withr_2.5.2       magrittr_2.0.3    digest_0.6.33     grid_4.3.1       
## [33] rstudioapi_0.15.0 hms_1.1.3         lifecycle_1.0.4   vctrs_0.6.4      
## [37] evaluate_0.23     glue_1.8.0        farver_2.1.1      ragg_1.2.6       
## [41] fansi_1.0.5       colorspace_2.1-0  rmarkdown_2.29    tools_4.3.1      
## [45] pkgconfig_2.0.3   htmltools_0.5.8.1 ggridges_0.5.6
```
